# A Randomized controlled trial of the Effect of intraVenous iron on Anaemia in Malawian Pregnant women (REVAMP): Statistical analysis plan

**DOI:** 10.12688/gatesopenres.13457.1

**Published:** 2021-12-07

**Authors:** Rebecca Harding, Ricardo Ataide, Martin N Mwangi, Julie A Simpson, Glory Mzembe, Ernest Moya, Zinenani Truwah, Brains Changaya Nkhwazi, Mphatso Mwabinga, William Nkhono, Kamija S Phiri, Sant-Rayn Pasricha, Sabine Braat

**Affiliations:** 1 Population Health and Immunity Division, The Walter and Eliza Hall Institute of Medical Research, 1G Royal Parade, Parkville, Melbourne, VIC, 3052, Australia; 2 Department of Infectious Diseases, Melbourne Medical School, The University of Melbourne, Melbourne, VIC, 3052, Australia; 3 Training and Research Unit of Excellence (TRUE), 1 Kufa Road, P.O. Box 30538, Chichiri, Blantyre, BT3, Malawi; 4 School of Public Health and Family Medicine, Department of Public Health, College of Medicine, University of Malawi, Private Bag 360, Chichiri, Blantyre, BT3, Malawi; 5 Centre for Epidemiology and Biostatistics, Melbourne School of Population and Global Health, University of Melbourne, Melbourne, VIC, 3052, Australia; 6 Diagnostic Haematology and Clinical Haematology, The Royal Melbourne Hospital and The Peter MacCallum Cancer Centre, Parkville, Melbourne, VIC, 3050, Australia; 7 Department of Medical Biology, Faculty of Medicine, Dentistry and Health Sciences, The University of Melbourne, Melbourne, VIC, 3050, Australia

**Keywords:** anaemia, iron deficiency, intravenous iron, ferric carboxymaltose (FCM), pregnancy, randomized controlled trial, statistical analysis, Malawi

## Abstract

**Background: **Anaemia affects more than half of Africa’s pregnancies. Standard care, with oral iron tablets, often fails to achieve results, with compliance and gastrointestinal side-effects being a significant issue. In recent years, intravenous iron formulations have become safe, effective, and quick to administer, allowing the complete iron requirements of pregnancy to be provided in one 15-minute infusion. The Randomized controlled trial of the Effect of intraVenous iron on Anaemia in Malawian Pregnant women (REVAMP) will evaluate whether a modern intravenous iron formulation, ferric carboxymaltose (FCM), given once during the second trimester is effective and safe in improving maternal and neonatal outcomes for treatment of moderate to severe anaemia in sub-Saharan Africa.

The objective was to publish the detailed statistical analysis plan for the REVAMP trial prior to unblinding the allocated treatments and performing the analysis.

**Methods: **REVAMP is a multicentre, two-arm, open-label, parallel-group randomized control trial (RCT) in 862 pregnant women in their second trimester. The trial statistician developed the statistical analysis plan in consultation with the trial management team based on the protocol, data collection forms, and study outcomes available in the blinded study database.

**Results:** The detailed statistical analysis plan will support the statistical analyses and reporting of the REVAMP trial after unblinding the treatment allocations.

**Conclusions:** A statistical analysis plan allows for transparency as well as reproducibility of reporting and statistical analyses.

## Introduction

Approximately 36.5% of pregnant women globally are anaemic ( World Health Organization, 23 April 2021), and iron-deficiency anaemia (IDA) is the cause of almost half of all anaemia during pregnancy ^1^. In sub-Saharan Africa, 46% of all pregnant women are anaemic ^1^. The adverse outcomes of anaemia during pregnancy extend to both the mother – including the life-threatening complication of postpartum haemorrhage – and the baby – including prematurity, low birth weight ^2^, impaired development ^3^, and increased mortality ^4, 5^. Thus, reducing the burden of anaemia in women is one of the key World Health Organization (WHO) 2025 global nutrition targets ^6^.

Oral iron is the established approach for preventing and treating IDA in pregnancy and infancy ^7^. However, oral iron may be poorly tolerated due to gastrointestinal adverse events ^8^ and poorly adhered to over an entire course of treatment. This may result in suboptimal adherence to prevention programs in low- and middle-income countries ^9, 10^. Over the past two decades, parenteral (intravenous) iron therapies have dramatically advanced in terms of safety and convenience, providing an alternative to oral therapy. Modern parenteral iron formulations are commonly used in high-income countries to treat IDA during pregnancy ^11^. Ferric carboxymaltose (FCM) is an established modern intravenous iron drug, which enables up to 1000 mg of elemental iron to be delivered in a single 15-minute infusion ( NPS Medicinewise, 01 May 2021). FCM is approved for use in pregnancy after the first trimester ^12^. The safety and convenience of FCM make this drug an exciting opportunity to treat anaemia in pregnancy in low-income countries. However, the evidence for the efficacy and safety of delivering FCM in pregnancy in low- to middle-income countries remains limited.

The Randomized controlled trial of the Effect of intraVenous iron on Anaemia in Malawian Pregnant women (REVAMP) is an open-label randomized controlled trial conducted in the Blantyre and Zomba districts of Malawi designed to determine the efficacy and safety of delivering FCM (compared with standard-of-care oral iron) in women with moderate or severe anaemia in the second trimester of pregnancy ^13^ ( ACTRN12618001268235). The primary outcome of the trial is maternal anaemia at 36 weeks’ gestation, and the key neonate outcome is birthweight. The trial recruited the first participant in November 2018 and completed follow-up to one-month postpartum in September 2021.

This paper describes the planned analysis for the REVAMP trial. This statistical analysis plan supersedes the plan provided in the trial registry and published protocol ^13^. Finalization of the statistical analysis plan before study unblinding has been undertaken to ensure transparency in the methods used to analyze and report the data and ultimately create the evidence for the effects of intravenous iron supplementation on recovery from prenatal anaemia, haemoglobin, iron status, postpartum haemorrhage, and delivery outcomes.

## Methods

The trial protocol is summarised elsewhere ^13^.

### Aims

The study’s main objective is to determine the efficacy and safety of a single intravenous iron administration during the second trimester of pregnancy – given as ferric carboxymaltose compared with routinely delivered oral iron – given as ferrous sulphate – in improving maternal (primarily anaemia) and neonatal (e.g., birth weight) outcomes.

### Design

**Figure 1.**
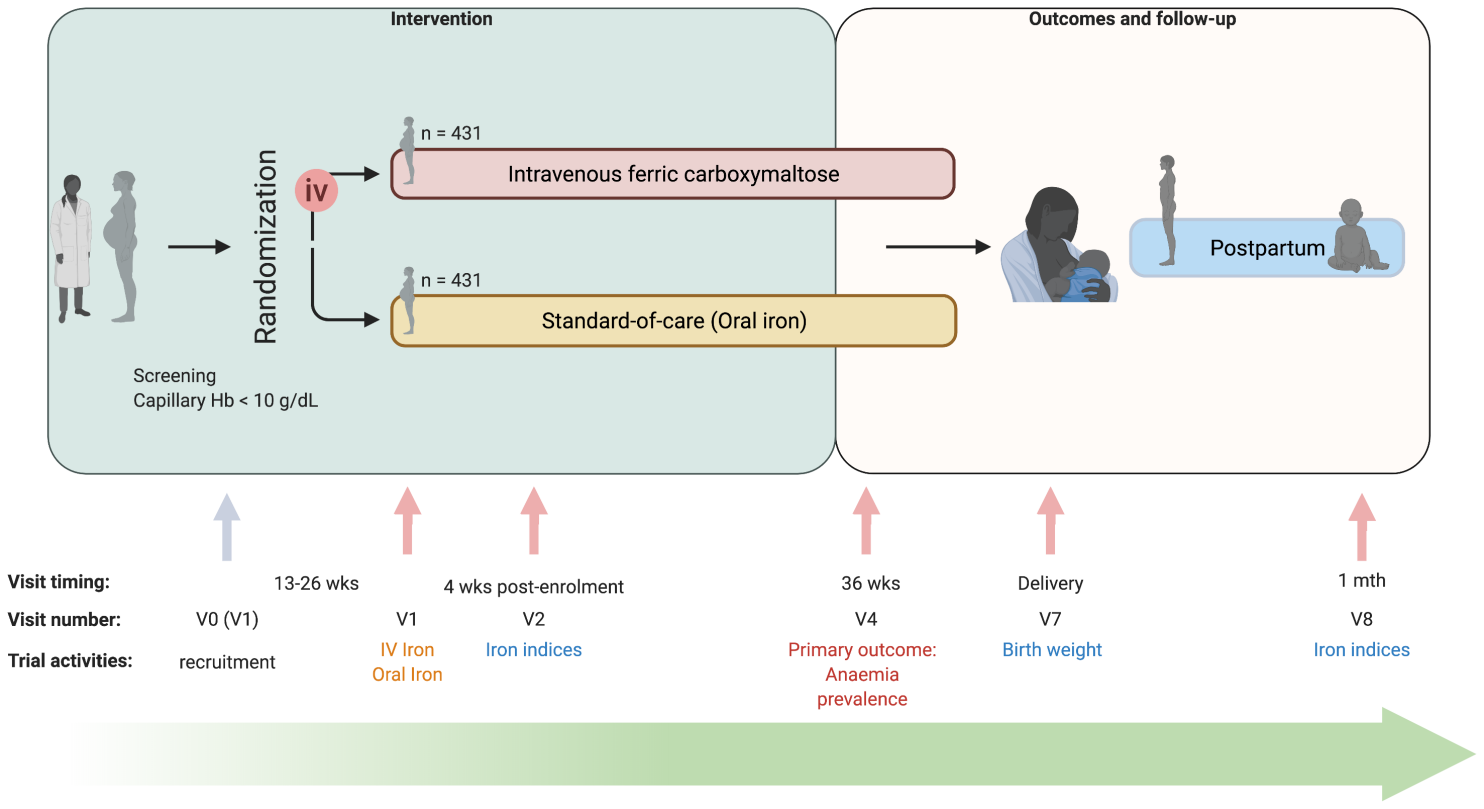
Study design and timeline of the Randomized controlled trial of the Effect of intraVenous iron on Anaemia in Malawian Pregnant women (REVAMP) (created with BioRender). V0 – visit 0; V1 – Visit 1; V2 – Visit 2; V4 – Visit 4; V7 – Visit 7; V8 – Visit 8; Abbreviations: iv – intravenous; wks – weeks. The study was designed as a two-arm trial (intravenous ferric carboxymaltose versus standard-of-care (oral iron)) where women were randomized in their second trimester of pregnancy. Study visits occurred over pregnancy, at birth, and follow-up to one-month postpartum. Women were scheduled to be visited in their home at 34 weeks’ gestation (Visit 3), and every two weeks from 38 weeks’ gestation until delivery (Visit 5 and 6) to measure capillary Hb, not included in the study design schema.

REVAMP is an open-label two-arm parallel-group randomized controlled trial in anaemic pregnant women (capillary haemoglobin <10 g/dL). Women were randomized to either IV ferric carboxymaltose 1000 mg (for women with weight >50 kg), or 20 mg/kg (for women with weight <50 kg) once during the second trimester; or oral iron 200 mg ferrous sulphate (approx. 65 mg elemental iron) twice daily for 90 days or the duration of pregnancy, whichever was shorter. Study visits occurred over pregnancy, at birth, and follow-up to one-month postpartum ( Figure 1). The study received ethics approval from The College of Medicine Research Ethics Committee (COMREC), Blantyre, Malawi (COMREC: P.02/18/2357) and the Walter and Eliza Hall Institute of Medical Research Human Ethics Committee, Melbourne, Australia (WEHI REC:18/02). It was prospectively registered at the Australian New Zealand Clinical Trials Registry ( ACTRN12618001268235, registered on 27 July 2018).

### Setting

The trial took place in southern Malawi at two sites: the coordinating site in Zomba Central Hospital, Zomba district, and a second site in Blantyre district at Limbe Health Centre. Both sites had all the resources required to recruit eligible participants, prepare, and administer the study drugs, monitor safety, treat adverse effects, and measure trial outcomes.

### Participants

Women eligible for enrolment were in their second trimester (between 13–26 weeks of gestation) and presented with a capillary haemoglobin level below 10 g/dL, as measured by HemoCue ^®^ Hb 301 system. In addition, participants were eligible if they were negative for malaria (determined using a rapid diagnostic test (RDT)), planned to deliver at the health facility and were able to provide written informed consent (or have a legal guardian do so if <18 years old). Women with clinical symptoms of infection, any severe condition requiring hospitalization, a history of pre-eclampsia, or known hypersensitivity to the study drugs were not eligible for recruitment.

### Randomization and treatment allocation

Women were randomly allocated to one of the two treatment arms with 1:1 allocation using a randomization schedule of randomly permuted blocks stratified by site to achieve balance between the arms within each site. The randomization list was computer-generated by an independent statistician and participants were randomly allocated using sealed, opaque envelopes. Although the trial is open-label, laboratory scientists measuring haemoglobin concentration, midwives collecting birth outcome data, and investigators and researchers in Australia (including data managers and statisticians in Melbourne) are blinded to the treatment allocation during the conduct of the trial until the database is locked and ready for unblinding.

### Outcome variables

 All efficacy and laboratory outcomes were measured at baseline, 28 days post randomization, 36 weeks’ gestation, delivery and one month postpartum for mothers and at delivery and one month postpartum for neonates. Data related to safety, including non-serious adverse events (AEs) and serious adverse events (SAEs), were collected across the total study period.

### Maternal

 The primary outcome of the study was maternal anaemia (defined as a venous haemoglobin concentration less than 11.0g/dL) at 36 weeks’ gestation.

 Secondary maternal outcomes included laboratory indices (haemoglobin and ferritin concentrations) and haematological and iron diagnoses (anaemia, moderate/severe anaemia, iron deficiency, iron deficiency anaemia) at 28 days post randomization, 36 weeks’ gestation, and one month postpartum. Haemoglobin concentration, anaemia and moderate/severe anaemia were also included at delivery. Using mother’s haemoglobin (g/dL), ferritin (ug/L), and C-reactive protein (CRP, mg/L), anaemia was defined as haemoglobin concentration less than 11.0 g/dL, moderate/severe anaemia as haemoglobin concentration less than 10.0 g/dL, iron-deficient as ferritin <15 mg/L adjusted for inflammation (CRP >5 mg/L), and iron deficiency anaemia as iron deficient and anaemic.

Maternal safety outcomes included reported adverse events (including serious adverse events defined as any adverse event that resulted in death, were life threatening, required either inpatient hospitalization or prolongation of hospitalization, resulted in a persistent or significant disability/incapacity or resulted in a congenital anomaly/birth disorder), all-cause sick visits (including specifically, visits due to clinical malaria), hypophosphatemia (mild: 0.64< phosphate (PO4) <0.80 mmol/L, moderate: 0.32<PO4<0.64 mmol/L, severe: PO4 <0.32 mmol/L), inflammation (elevated C-reactive protein), and severe medical events (includes haemorrhage, need for transfusion, ICU admission, mortality). Adverse events were coded using version 5.0 of the Common Terminology Criteria for Adverse Events (CTCAE) ( US Department of Health Human Services, 27 November 2020) and consisted of selecting an appropriate Preferred Term (PT) and System Organ Class (SOC) for each AE verbatim term. All AEs were coded on an ongoing basis by two physicians independent from each other and without knowledge of the treatment assignment (i.e., blinded). Any discrepancies regarding the selection of an appropriate PT or SOC will be resolved via discussion between the two physicians. All AE coding will be finalised before unblinding of the study database.

 Additional data collected included maternal baseline characteristics: age, parity, gravidity, anthropometry, religion, education, marital status, income source, recent malaria status, and HIV positivity status. 

### Neonate

The key neonatal outcome was birthweight, as measured in grams within 24 hours of birth.

 Secondary neonatal outcomes included birth length, gestation duration, and adverse birth outcomes (e.g., low birth weight, premature birth, small for gestation age, foetal loss – both individually and as a composite) within 24 hours of birth and growth (weight-for-age z-score, length-for-age z-score, weight-for-length z-score) and laboratory indices (haemoglobin concentration) at one month of age. Using gestational duration (weeks), premature birth was defined as a neonate born before 37 completed weeks of gestation, foetal loss was defined as pregnancy loss before 28 completed weeks’ gestation or stillbirth as the birth of a child showing no signs of life after 28 weeks’ gestation. Low birth weight was defined as a neonate born with a birthweight <2500 g. Small for gestational age was derived using the neonate’s gestation duration and birthweight together with the sex of the child according to INTERGROWTH-21 standards ^14^. Growth outcomes (z-scores) were derived using the neonate’s length and weight together with age and sex of the neonate according to age and sex specific WHO international growth standards ^15^.

 Neonate safety outcomes included (serious) adverse events, all cause sick visits (includes infection-related visits, diarrhoea-related visits, clinical malaria-specific visits).

### Sample size

 The sample size for the trial was to recruit 862 pregnant women (431 per arm) to have 80% power to detect that FCM will result in a 10% improvement in anaemia cure (a prevalence of 60% for standard oral therapy and 50% for FCM) ^2, 12, 16^, allowing for a two-sided alpha of 5% and a 10% loss to follow-up. The sample size also has at least 80% power to detect an absolute difference between standard-of-care oral iron and FCM of 100 g in the neonatal outcome of birth weight, assuming a standard deviation of 450 g and a two-sided alpha of 5%, similar to the effect size seen in a trial of Kenyan women receiving oral iron when compared with placebo ^17^. No interim analyses to stop the trial early were planned, and no interim analysis was conducted. 

### Statistical analysis plan

The analysis will be conducted by statisticians at The Walter and Eliza Hall Institute of Medical Research (WEHI), Melbourne, Australia. After all study data are available and clean, a blinded data review meeting to review protocol violations and missing data will be held prior to database lock. The final statistical analysis plan will be signed off during this meeting. Analysis will be conducted using Stata/SE (StataCorp. 2019. College Station, TX: StataCorp LLC). The analysis of the primary outcome will be independently checked. Discrepancies will be discussed and resolved by consensus. 

### General principles 

***Maternal*.** The intention-to-treat population will consist of all mothers who were randomized and included in the analysis of all primary and secondary maternal outcomes according to the randomized allocation. The per-protocol population will consist of all mothers who were randomized, and without protocol violations. A protocol violation is defined as no informed consent or violating inclusion/exclusion criteria (e.g., twin pregnancy). These protocol violations are based on pre-randomisation characteristics only and expected to be balanced between treatment groups. Data on adherence to standard-of-care is restricted due to the suspension of home visits ( Figure 1) to mitigate risks during the COVID-19 pandemic. The safety population will consist of all women who received at least one study treatment (either IV iron or standard-of-care) and included in the analyses of all safety maternal outcomes according to their actual treatment. Mothers who have withdrawn consent for use of all their data will be excluded from all analyses.

***Neonate*.** The intention-to-treat population will consist of all neonates of mothers who were randomized and included in the analysis of the key and secondary neonatal outcomes according to the mother’s randomized allocation. The per-protocol population will consist of all neonates born to mothers who were randomized, and without protocol violations. A protocol violation is defined as no informed consent or the mother being a protocol violation. The safety population will consist of all neonates born to mothers who received at least one treatment (either IV iron or standard-of-care) and included in the analyses of all safety neonatal outcomes according to the actual treatment of the mother. Neonates who have had consent withdrawn for use of all their data will be excluded from all analyses.

### Trial profile

**Figure 2.**
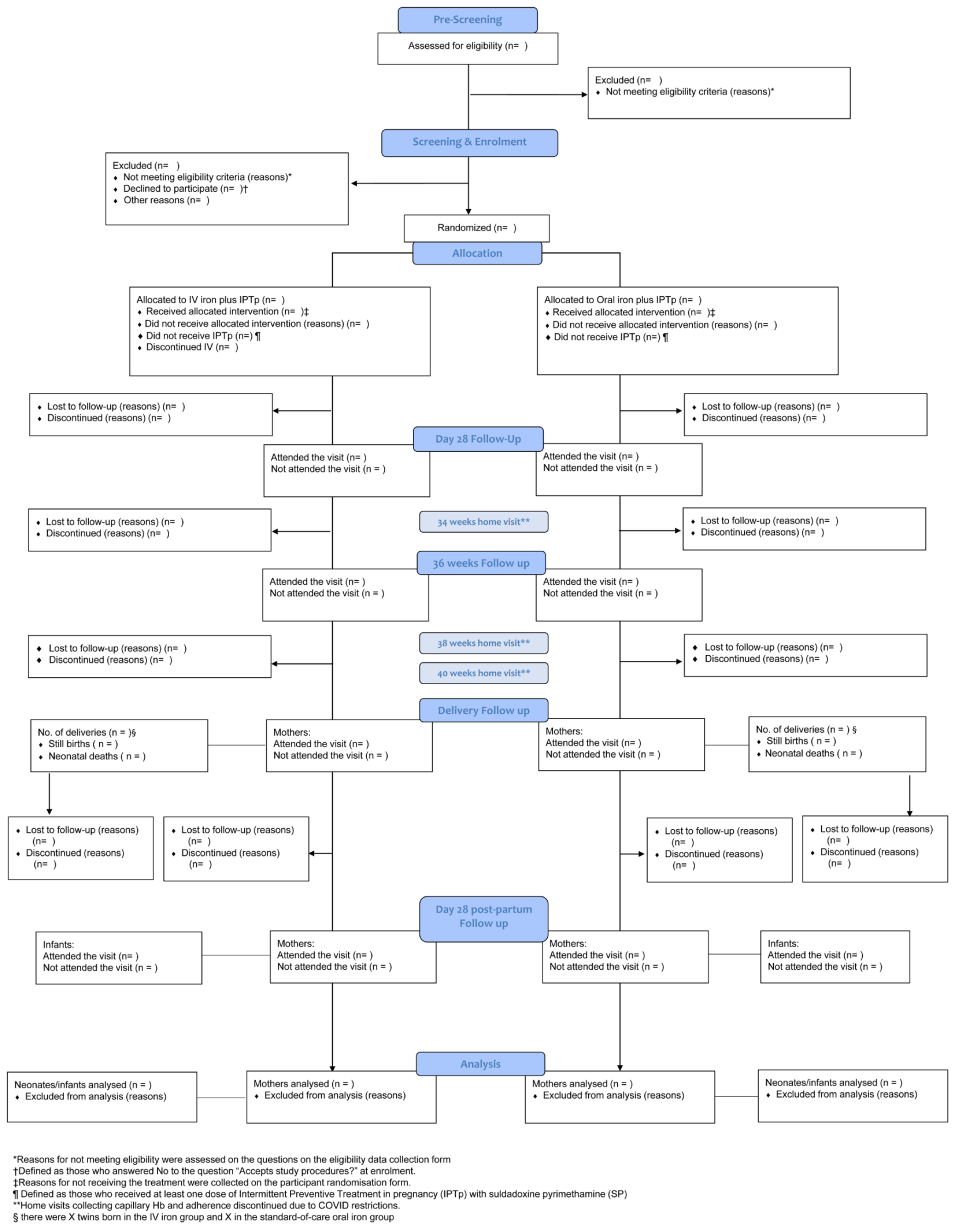
The CONSORT flow chart.

 The flow of mothers and their neonates through the trial will be presented in a Consolidated Standards of Reporting Trials (CONSORT) diagram, reasons for exclusion will be reported ( Figure 2).

### Baseline characteristics

Maternal demographic and baseline variables will be summarised descriptively and presented by treatment group. No formal comparisons will be made between treatment arms. Characteristics will be summarised using frequencies and percentages (based on the non-missing sample size) for categorical variables, mean and standard deviation for continuous variables, or median and quartiles (25th and 75th percentile) for non-normally distributed continuous variables. 

### Efficacy outcomes: analysis

***Maternal*.** Maternal anaemia (primary outcome) at 28 days post randomization, 36 weeks’ gestation (primary timepoint), delivery and 28 days postpartum will be analyzed using a log-binomial regression model, including mothers as a random intercept to account for multiple time points, and adjusted for the stratification variable used during the randomization (site). The model will include the standard-of-care oral iron group as the reference group. The treatment effect will be estimated from this model as the prevalence ratio of IV iron versus standard-of-care oral iron. In case of non-convergence, we will fit a modified Poisson regression model with robust error variance, including mothers as a random intercept to account for the multiple timepoints.

Secondary repeated time-point binary outcomes (moderate/severe anaemia, iron deficiency, iron deficiency anaemia) will be analyzed similarly to anaemia at 28 days post randomization, 36 weeks’ gestation, delivery (for moderate/severe anaemia), and 28 days postpartum.

Secondary repeated time-point continuous outcomes (haemoglobin concentration and ferritin concentration) will be analyzed using a likelihood-based longitudinal data analysis model ^18^. The model will assume a common baseline mean across the two treatment arms and an unstructured variance-covariance among the repeated measurements. The model will incorporate time point (study visit) as a categorical variable, treatment and treatment by study visit interaction and adjust for the stratification factor (site) as main effects. In case of non-convergence, we will consider alternative structures (first-order autoregressive, Toeplitz, compound symmetry). The treatment effect will be estimated from this model as the mean change from baseline to 28 days post randomization, 36 weeks’ gestation, delivery, and 28 days postpartum respectively between IV iron and standard-of-care oral iron. Ferritin (ug/L) will be log _e_ transformed before analysis, with the treatment effect expressed as a geometric mean ratio.

***Neonate*.** Birthweight will be analyzed by fitting a linear regression model, adjusting for the stratification factor (site). The treatment effect will be estimated from this model as the absolute difference in mean birth weight between IV iron and standard-of-care oral iron.

Secondary single time point continuous outcomes (gestation duration and birth length within 24 hours of birth, and haemoglobin concentration, length-for-age z-score, weight-for-age z-score, and weight-for-length z-score at one month of age) will be analyzed similarly to birthweight. Appropriate transformations may be applied to the variables before fitting the model if considered skewed. 

Secondary single time point binary outcomes (composite adverse birth outcome, low birth weight, foetal loss, premature birth, and small for gestational age) will be analyzed using a log-binomial regression model, adjusting for the stratification factor (site). The treatment effect will be estimated from this model as the risk ratio of IV iron versus standard-of-care oral iron. In case of non-convergence, we will fit a modified Poisson regression model with robust error variance.

### Safety outcomes: analysis

***Maternal*.** The number and percentage of women who died, reported at least one serious adverse event (including within 24 hours of randomization, within 14 days of randomization, antenatal and postpartum), reported at least one adverse event (including within 24 hours of randomization, within 14 days of randomization, antenatal and postpartum), who had at least one severe medical event (composite, and its components haemorrhage, need for transfusion, ICU admission, or mortality), had at least one common AE (>5% in any group), had at least one AE by system organ class and had at least one AE by preferred term will be reported and compared between treatment arms using a log-binomial regression model. In case of non-convergence for a safety outcome(s), a Poisson model with robust standard errors will be used to analyze the data. In addition, infusion-related adverse events will be reported separately for the IV iron group only.

The number and percentage of women with unplanned clinic visits (including all-cause sick visits and clinical malaria specific visits) and safety biomarkers (including inflammation and malaria RDT positive) will be reported and compared between treatments (by timepoint) using a log-binomial regression model. In case of non-convergence, a Poisson model with robust standard errors will be fitted to the data. The number and percentage of women with the safety biomarker hypophosphatemia will be reported and compared between treatments (by timepoint) using an ordered logistic regression model.

***Neonate*.** The number and percentage of neonates who died, had at least one serious adverse event, had at least one adverse event, had at least one common AE (>5% in any group), had at least one AE by system organ class and had at least one AE by preferred term will be reported and compared between treatments using a log-binomial regression model. In case of non-convergence, a Poisson model with robust standard errors will be used.

The number and percentage of neonates with unplanned clinic visits (including all-cause sick visits, diarrhoea related visits, respiratory related visits and clinical malaria specific visits) will be reported and compared between treatments using a log-binomial regression model. As for other binary outcomes, a Poisson model with robust standard errors will be fitted if there are non-convergence issues when fitting a log-binomial regression model.

### Reporting and methods for missing data

To describe the missing data, the frequency and percentage of study participants with missing data at baseline, 28 days post randomization, 36 weeks’ gestation, delivery and one month postpartum will be summarised for anaemia (mothers) and birth weight (neonates) by treatment group. In addition, baseline and demographic characteristics will be summarised by those with baseline only, incomplete data at any visit, and complete data at all visits for anaemia (mothers) to explore the missing data assumption(s) and identify any study variables not included in the target analyses that are potentially associated with missing/not missing of these study variables (known as auxiliary variables). As a rule of thumb, if the proportion of missing data is below approximately 5%, those values will be considered negligible in the case of maternal anaemia or live-born neonates with missing birth weight ^19^.

For dealing with missing data in the analyses of primary and secondary outcomes, the primary analysis will be an available case analysis performed for repeated time point outcomes (e.g., anaemia) and a complete case analysis for single time point outcomes (e.g., birth weight).

***Maternal*.** As the primary strategy to handle missing data, the analysis of maternal anaemia (repeated assessments) will use a likelihood-based approach. This approach relies on the underlying assumption that the probability of missing outcome data is not related to the missing data after conditioning on observed data in the model (Missing at Random [MAR]).

If the missing data is not negligible, additional analysis will be performed whereby missing maternal haemoglobin data will be multiply imputed using chained equations, separately by treatment group. The imputation model will include site, parity, gestational age at baseline and body mass index (BMI).

In addition, auxiliary variables identified during the blinded data review meeting may be included. Maternal haemoglobin will be imputed using a linear regression model. The missing outcome data at 28 days post randomization, 36 weeks’ gestation, delivery and one month postpartum will be imputed using the “just another variable” approach (also known as imputing in wide format), which requires a separate imputation model for imputing the variable at each assessment time ^20^. The number of imputed data sets will be greater than or equal to the percentage of missing data in the available case analyses. Using these imputed data sets, an analysis based on a pattern-mixture model ^21^ consisting of applying a delta-adjustment to the imputed values by treatment group will be conducted. Within the standard-of-care group participants with missing data will be assumed having both a poorer and better response than those with observed data while no a priori difference is anticipated in the mean response for the IV iron group. Differences in baseline participant characteristics between those with and without data will inform delta-values to explore in both treatment groups. After deriving maternal anaemia from the imputed haemoglobin values, the imputed data sets will be analyzed using a log-binomial regression model. The estimates from the analyses of the imputed data sets will be combined to obtain a pooled common estimate and corresponding confidence interval for the effect of the iron intervention on maternal anaemia using Rubin’s rules. The delta-adjustment method within the multiple imputation framework assumes a Missing Not At Random (MNAR) assumption for the outcome.

***Neonate*.** The analysis of birthweight will use a complete-case analysis among those live-born. This approach relies on the underlying assumption that the probability of missing outcome data is not related to the observed or missing data (Missing Completely at Random [MCAR]).

If the missing data is not negligible, additional analysis will be performed whereby missing birthweight data will be multiply imputed, separately by treatment group. The imputation model will include site, sex, gestational age at baseline and maternal BMI. In addition, auxiliary variables identified during the blinded data review meeting may be included. Birthweight will be imputed using a linear regression model. The number of imputed data sets will be greater than or equal to the percentage of missing data in the complete case analyses. The estimates from the analyses of the imputed data sets will be combined to obtain a pooled common estimate and corresponding confidence interval for the effect of the iron intervention on birthweight using Rubin’s rules. This approach relies on the MAR assumption for the outcome, birthweight.

### Additional analyses 

***Maternal*.** In addition to the analysis model for all maternal efficacy outcomes adjusted for site as a main effect, additional analyses will be performed for these outcomes:

1. Analyses consisting of models adjusted for auxiliary variables:
  a. Adding to the model adjusted for site, the main effect of parity (primiparous vs. multiparous), gestational age at baseline (continuous), and BMI at baseline (continuous).
  b. Adding to the model adjusted for site, parity, gestational age at baseline and BMI (except for anaemia, ferritin and iron deficiency), the main effects of inflammation status at baseline, iron-deficient status at baseline, haemoglobin at baseline (continuous), HIV positive status at baseline, and re-screened post-previous positive malaria RDT status.
  c. Adding to the model adjusted for site, the main effect of variables in demographic and/or baseline characteristics demonstrating an imbalance between treatment arms after unblinding.

2. Analysis of the model adjusted for site for the per-protocol population.
3. Analysis of the model adjusted for site for the per-protocol population adjusted for baseline characteristics considered not balanced between the arms for the per-protocol population.

Furthermore, we will report the number-needed-to-treat (NNT) and 95% confidence interval for maternal anaemia at 36 weeks’ gestation. 

***Neonate*.** In addition to the analysis model for all neonate efficacy outcomes adjusted for site as a main effect, additional analysis will be performed for these outcomes:

1. Analyses consisting of models adjusted for auxiliary variables:
  a. Adding to the model adjusted for site, the main effect of sex of the infant (female or male), gestational age at baseline (continuous), and maternal BMI at baseline (continuous).
  b. Adding to the model adjusted for site, sex, gestational age at baseline and maternal BMI the main effect of maternal haemoglobin at baseline (continuous).
  c. Adding to the model adjusted for site, the main effect of variables in demographic and/or baseline characteristics demonstrating imbalance between treatment arms after unblinding.

2. Analysis of the model adjusted for site for the per-protocol population.
3. Analysis of the model adjusted for site for the per-protocol population adjusted for baseline characteristics considered not balanced between the arms for the per-protocol population.

Furthermore, we will report the NNT and 95% confidence interval for low birthweight. 

### Multiple testing

 No adjustment for multiplicity is planned for the primary maternal outcome (anaemia at 36 weeks’ gestation) and key neonate outcome (birth weight). We will test the primary null hypothesis of no difference between IV iron and standard-of-care oral iron at a two-sided 5% level of significance. Estimates and two-sided confidence intervals will be presented, along with multiplicity unadjusted P-Values.

 The Holm procedure ^22^ will be used to ensure control of the Type I error rate for secondary maternal outcomes at 36 weeks’ gestation (haemoglobin concentration, moderate/severe anaemia, ferritin, iron deficiency, and iron deficiency anaemia) and neonate outcomes (gestation duration, birth length, composite adverse birth outcome within 24 hours of birth and infant growth at 1-month postpartum) separately. We will present multiplicity unadjusted P-values along with the estimate and 95% confidence intervals and footnote the comparisons meeting the statistical significance threshold according to the Holm procedure.

No multiplicity adjustment is planned for other secondary outcomes at 28 days post randomization, delivery, and 28 days postpartum. We will present the estimate and two-sided 95% confidence interval and no P-Values will be presented. We will present the multiplicity unadjusted P-Values for the safety outcomes; no multiple testing adjustment is planned.

### Subgroup analyses

 Exploratory subgroup analyses will be performed for the outcomes of maternal anaemia at 36 weeks’ gestation, haemoglobin concentration at 36 weeks’ gestation, birth weight, low birth weight, gestation duration, and premature birth. The following subgroups will be explored: parity (primiparous vs. multiparous), baseline HIV status (positive vs negative), baseline severe anaemia status (yes vs no severe anaemia), baseline iron-deficient status (yes vs no ID), baseline iron-deficient anaemia status (yes vs no IDA), baseline inflammation status (yes vs no elevated CRP), re-screened after positive malaria RDT at pre-screening (yes vs no) and site (Blantyre, Zomba). In addition, subgroup (main effect) and the subgroup-by-treatment interactions term will be added to the unadjusted model to evaluate whether the treatment effect (IV iron versus standard-of-care) differs between subgroup categories. No multiplicity adjustments are planned for the subgroup analyses due to their explorative nature. Results of the subgroup analyses (effect estimate and 95% Confidence Interval) will be displayed using Forest plots.

### Trial status

This statistical analysis plan is an extension of the REVAMP protocol ^13^ and documents version 1 dated October 26, 2021. Any changes to this version between publishing and unblinding will be tracked and still considered as planned analyses. The statistical analysis plan will be approved during the blinded data review before breaking the allocation code, after which any changes after will be considered post-hoc.

## Discussion

Antenatal anaemia remains a significant public health concern in low-to-middle income countries. Although oral iron supplementation remains a cheap formulation, suboptimal adherence and common limiting gastrointestinal adverse effects from the drugs may limit effectiveness. If our data demonstrate a benefit from intravenous iron on maternal outcomes and potentially also on critical neonatal outcomes such as birth weight, the findings will provide evidence for the beginning of a clinical rationale for developing strategies for implementing this intervention in practice. Thus, results from this trial could ultimately transform the way anaemia is treated in low-income settings and have long term benefits for maternal and child health, ultimately resulting in benefits for maternal and child survival.

## Data availability

No data are associated with this article.
