# Supplementary material for: A Randomized controlled trial of the Effect of intraVenous iron on Anaemia in Malawian Pregnant women (REVAMP): Statistical analysis plan
Source: Gates Open Res. 2022 Apr 14;5:174. Originally published 2021 Dec 7. [Version 2] doi: 10.12688/gatesopenres.13457.2 (PMC9019159; doi:10.12688/gatesopenres.13457.2)
Supplement: Peer review history

# Peer review: Reviewer response for version 1

**DOI:** 10.21956/gatesopenres.14714.r31529

**Published:** 2022-01-04

**Authors:** Tapan Rai

**Affiliations:** 1 School of Mathematical and Physical Sciences, University of Technology Sydney, Sydney, NSW, Australia

This is a well-written statistical analysis plan which is written as concisely as possible. Although the publication of statistical analysis plans for clinical trials has become more common over the past few years, it is not yet standard practice and the art of writing a plan for publication as a journal article is still evolving. This article does a very good job of this and may be used as a guide by other research teams. Having said that, I believe that there may be some places where the authors could clarify a few points - especially since other teams are likely to look at this plan as a model in the future:

1. The Intention-to-Treat (ITT) and per-protocol cohorts could be better defined. I would specifically seek clarification of the following points:
  1. The authors state: "The per-protocol population will consist of all mothers who were randomized, and without protocol violations. A protocol violation is defined as no informed consent ...". This seems to make it clear that participants who did not provide informed consent will be excluded from the per-protocol cohort. However, it also seems to suggest that they will be included in the ITT cohort. This is not usually the case, since the trial should not generally be collecting data from participants who have not or are unable to provide informed consent - unless informed consent is provided by proxy (e.g. by next-of-kin). If the authors did not intend to suggest that participants without informed consent are to be included in the ITT cohort, please reword or restate the definition of a protocol violation so as to remove any doubt about this. Alternatively, can the authors, please, clarify whether:
    1. The trial collected data from participants without informed consent?
    2. If so, was consent obtained from next-of-kin?
    3. Are participants who did not provide informed consent to be included in the ITT cohort? 
  2. If a participant refused treatment after being randomised or were otherwise not given the allocated treatment in the expected timeframe, they would normally be analysed in the group to which they are randomised in an ITT analysis. However, it is not clear how these participants would be analysed in the per-protocol analysis - would they be deleted from the analysis or analysed with the standard care group (e.g. if they were randomised to the FCM group but received standard care)?
2. The proposed log-binomial models (with possible use of a Poisson model with robust errors, if the log-binomial model fails to converge) are appropriate in this situation. However, since statistical analysis plans are often read by clinical researchers (who are not all statisticians), please write 1-2 sentences explaining your rationale for selecting this approach rather than a logistic regression model which is more common (and more clinical researchers are likely to be familiar with). My hope is that this will provide some guidance/insight on this to other researchers reading this statistical analysis plan.
3. The statistical analysis plan for secondary repeated timepoint continuous outcomes, mentions the use of the interaction effect between treatment group and timepoint (p. 7, Paragraph 3). This is standard practice to assess between-group differences at specific timepoints in a longitudinal model. I expect that you are using a similar approach (interaction between treatment group and timepoint) for the primary and secondary repeated timepoint binary outcomes as well. However, you have not mentioned this in the text. For consistency and clarity, please state this clearly when outlining the analysis for these binary outcomes as well (alternatively, if you are not using some other approach that does not require the interaction effect in these models, please specify this and elaborate on this so that it is clear to the reader).
4. The description for the reporting and analysis of adverse events needs some clarification. There are several adverse event outcomes mentioned (for maternal adverse events). These include the number and percentage of women who:
  1. died,
  2. reported at least one serious adverse event (including within 24 hours of randomization, within 14 days of randomization, antenatal and postpartum),
  3. reported at least one adverse event (including within 24 hours of randomization, within 14 days of randomization, antenatal and postpartum),
  4. at least one severe medical event (composite, and its components
    haemorrhage, need for transfusion, ICU admission, or mortality),
  5. had at least one common AE ,
  6. had at least one AE by system organ class and had at least one AE by preferred term will be reported.

This suggests that these will be reported separately for each stated timepoint (e.g. at least one AE within 24 hours, at least one AE in the period 24 hours-14 days post randomization etc.) and by organ system etc. Please clarify if this is the case, and, if this is the case, please clarify what response variable will be used for the log-binomial model associated with adverse events - is it one model for each AE outcome mentioned above (which is not likely to converge if the number of events in each category is small) or is there some designated response variable to be used?

 ​​​​​​​

 Overall, it may be easier to clarify how the adverse events would be reported by providing sample tables of the reporting of descriptive statistics as an appendix. Providing such sample tables would also help make clearer how some of the other descriptive statistics will be reported.

Is the study design appropriate for the research question?

Yes

Is the rationale for, and objectives of, the study clearly described?

Yes

Are sufficient details of the methods provided to allow replication by others?

Yes

Are the datasets clearly presented in a useable and accessible format?

Not applicable

Reviewer Expertise:

Biostatistics

I confirm that I have read this submission and believe that I have an appropriate level of expertise to confirm that it is of an acceptable scientific standard.

# Response

**Published:** 2022-04-07

**Authors:** Rebecca Harding

**Affiliations:** The Walter and Eliza Hall Institutue of Medical Research, Australia

Thank you for your review and feedback comments

 Responses to your minor comments:

 1.

 1. Thank you for pointing this out. We did not intend to suggest that participants without informed consent are to be included in the ITT population. The definition has been re-worded in the general principles section:

 “A protocol violation is defined as those who have **withdrawn informed consent for the use of all their data** or violating inclusion/exclusion criteria (e.g., twin pregnancy).”

 To clarify: The trial did not collect data from participants without informed consent. Participants who did not provide informed consent will not be included in the ITT population.

 2. Thank you for pointing this out. The following text has been added in the general principles section.

 Maternal - Mothers who are found to be non-adherent to treatment (e.g., refused or not provided treatment after randomisation) will be excluded from the per-protocol population analysis.

 Neonate - Neonates of mothers who are found to be non-adherent to treatment (e.g., refused or not provided treatment after randomisation) will be excluded from the per-protocol population analysis.

 2.

 The following text has been added to the efficacy outcomes analyses section.

 We have selected the prevalence ratio as an effect measure because its interpretation as the ratio change in prevalence is easier to understand than the interpretation of the odds ratio for clinical researchers.  

 3.

 Thank you for pointing this out, yes, we are using a similar approach of a treatment and treatment by study visit interaction for the primary repeated timepoint binary outcomes as well.

 The following bold text has been added to the efficacy outcomes: analyses section:

 Maternal anaemia (primary outcome) at 28 days post randomization, 36 weeks’ gestation (primary timepoint), delivery and 28 days postpartum will be analyzed using a log-binomial regression model, including mothers as a random intercept to account for multiple time points **. The model will include a treatment and treatment by study visit interaction and** adjust for the stratification variable used during the randomization (site).

 4.

 The analysis will be performed by adverse event outcome with the response variable being the binary adverse event outcome (e.g. died: yes/no, had at least one common AE: yes/no). We have changed and added the following bold text to the safety outcomes: analyses section:

 The number and percentage of women who died, reported at least one serious adverse event ( **overall, and for each of the following time intervals: **within 24 hours of randomization, within 14 days of randomization, antenatal and postpartum), reported at least one adverse event ( **overall, and for each of the following time intervals: ** within 24 hours of randomization, within 14 days of randomization, antenatal and postpartum), who had at least one severe medical event (composite, and its components haemorrhage, need for transfusion, ICU admission, or mortality), had at least one common AE (>5% in any group), had at least one AE by system organ class and had at least one AE by preferred term will be reported. **Each of the above listed adverse event outcomes will be** compared between treatment arms using a log-binomial regression model. In case of non-convergence for a safety outcome(s), a Poisson model with robust standard errors will be used to analyze the data. **Prevalence ratios and 95% confidence intervals will be presented for  IV iron versus standard-of-care oral iron.** In addition, infusion-related adverse events will be reported separately for the IV iron group only.

 Thank you for your suggestion of providing sample tables in an appendix. We hope to have satisfactory clarified our approach with regards to the adverse event safety outcomes in text.

# Peer review: Reviewer response for version 1

**DOI:** 10.21956/gatesopenres.14714.r31557

**Published:** 2021-12-23

**Authors:** Mavuto Mukaka

**Affiliations:** 1 Mahidol Oxford Tropical Medicine Research Unit, Faculty of Tropical Medicine, Mahidol University, Bangkok, Thailand; 2 University of Oxford, Oxford, UK

The Statistical analysis plan has been well constructed with excellent statistical methodology. There are a few areas that need to be addressed to improve the plan as follows:

**Minor comments:**

1. The plan clearly defines the Intention to treat and per protocol populations, however, there is no commitment in the plan as to which of the two approaches will form the primary strategy for the primary outcome. Is it the ITT or the PP? This should be clarified. Simply defining intention to treat or per-protocol populations without further specification is not sufficient.
2. The plan is clear that for the primary outcome, a log-binomial regression model will be used or in case of non-convergence, a modified Poisson regression model with robust error variance will be fitted. However, the measure of effect has not been clearly stated. For a non-technical audience, it is important to state the measure of effect like how the authors have stated for neonatal birth weight. 
3. In the Trial status section, authors need to state about signing of the final version of SAP by the investigators.
4. The authors state that if the proportion of missing data will be less than 5%, available case and complete case analysis approaches will be used. While I agree with this rule of thumb, I am wondering how authors will handle the intention to treat analyses if there will be less than 5% missing outcome data. Will they analyse fewer number of participants than randomized? If so what will be the implications of the ITT principle?
5. Will ”mixed effects” be considered in any of the specified statistical models? The authors should state in the SAP if these are anticipated.
6. The CONSORT flow chart has been very well constructed and is clear. However, there is no indication on which levels constitute ITT analyses and which ones constitute PP analyses.
7. In the sample size section, the software that was used for sample size calculations should be stated.
8. On page 4, the authors state that “The primary outcome of the study was maternal anaemia (defined as a venous haemoglobin concentration less than 11.0g/dL) at 36 weeks’ gestation.” It would be good to insert the reference for the cut-off point.

Is the study design appropriate for the research question?

Yes

Is the rationale for, and objectives of, the study clearly described?

Yes

Are sufficient details of the methods provided to allow replication by others?

Yes

Are the datasets clearly presented in a useable and accessible format?

Not applicable

Reviewer Expertise:

Statistics, epidemiology, clinical trials

I confirm that I have read this submission and believe that I have an appropriate level of expertise to confirm that it is of an acceptable scientific standard.

# Response

**Published:** 2022-04-07

**Authors:** Rebecca Harding

**Affiliations:** The Walter and Eliza Hall Institutue of Medical Research, Australia

Thank you for your review and feedback comments

 Responses to your minor comments:

 1.

 Thank you for pointing this out. This has been clarified in the general principles section. The following bold text has been added:

*Maternal*. **Primary analyses will be undertaken on an intention-to-treat basis.** The intention-to-treat population will consist of all mothers who were randomized…

*Neonate*. **Primary analyses will be undertaken on an intention-to-treat basis. **The intention-to-treat population will consist of **all live-born **neonates **(with the exception of the stillbirth outcome)** of mothers who were randomized and included in the analysis of the key and secondary neonatal outcomes according to the mother’s randomized allocation.

 2.

 This point has been addressed in the efficacy outcomes: analysis section. The following bold text has been added:

 “In case of non-convergence, we will fit a modified Poisson regression model with robust error variance, including mothers as a random intercept to account for the multiple timepoints. **The treatment effect will be estimated from this model as the prevalence ratio of IV iron versus standard-of-care oral iron**.”

 3.

 This point has been addressed and the following bold text has been added in the trial status section:

 “The **final** statistical analysis plan will be approved during the blinded data review **and** **signed** before breaking the allocation code, after which any changes will be considered post-hoc.”

 4.

 If the analysis of the primary maternal endpoint (anaemia at 36 weeks’ gestation) or the key neonate endpoint (birthweight) result in exclusion of participants due to missing all outcome data, then we will refer to this analysis as a modified intention-to-treat analysis. The following bold text has been added to the reporting and methods for missing data section:

 As a rule of thumb, if the proportion of missing data is below approximately 5%, those values will be considered negligible in the case of maternal anaemia or live-born neonates with missing birth weight [19] **, in which case we will refer to the analysis as a modified intention-to-treat analysis**

 5.

 The specified log-binomial regression model for repeated timepoint binary outcomes and the specified likelihood-based longitudinal data analysis model for repeated time point continuous outcomes contain both fixed and random effects.

 6.

 A footnote has been added to the CONSORT flow chart to indicate those who were included in the Intention-to-Treat (ITT) population.

 7.

 The following text has been added in the sample size section:

 “Sample size calculations were performed using Stata/SE (StataCorp. 2019. College Station, TX: StataCorp LLC). ”

  8.

 The reference for the cut-off point has been added.

 World Health Organization. Haemoglobin concentrations for the diagnosis of anaemia and assessment of severity. Vitamin and Mineral Nutrition Information System. Geneva, World Health Organization, 2011 (WHO/NMH/NHD/MNM/11.1) (http://www.who.int/vmnis/indicators/haemoglobin. pdf, accessed 17 Feb 2022).
